# Reducing the Nonindicated Use of IV Hydralazine in the Inpatient Setting

**DOI:** 10.1016/j.jaccas.2025.105712

**Published:** 2025-10-07

**Authors:** Ashwin Kumar, John Lynch, Jennifer Baldwin

**Affiliations:** Department of Medicine, University of Connecticut Health Center, Farmington, Connecticut, USA

**Keywords:** health care economics, hemodynamics, hypertension

## Abstract

**Background:**

Elevated blood pressure (BP) affects up to 72% of hospitalized patients. Intravenous (IV) antihypertensives are indicated for hypertensive emergencies, but they are often given for asymptomatic BP elevations, leading to hypotension, acute kidney injury, and intensive care unit transfers without improving outcomes.

**Project Rationale:**

At UConn John Dempsey Hospital, 69% of IV hydralazine use between June 2023 and January 2024 lacked appropriate indications.

**Project Summary:**

A redesigned order set required prescribers to select a guideline-supported indication before ordering hydralazine. The order also displayed the last 5 BPs and included hyperlinks to American Heart Association guidelines. Education sessions for residents and nurses reinforced best practices and provided alternatives for asymptomatic BP elevations.

**Take-Home Messages:**

Nonindicated IV hydralazine use decreased from 69% to 25%. Order set modifications had a greater impact than education alone. Embedding decision support with targeted education offers a scalable, reproducible model for safer inpatient prescribing.

## Graphical Abstract

**Figure:**
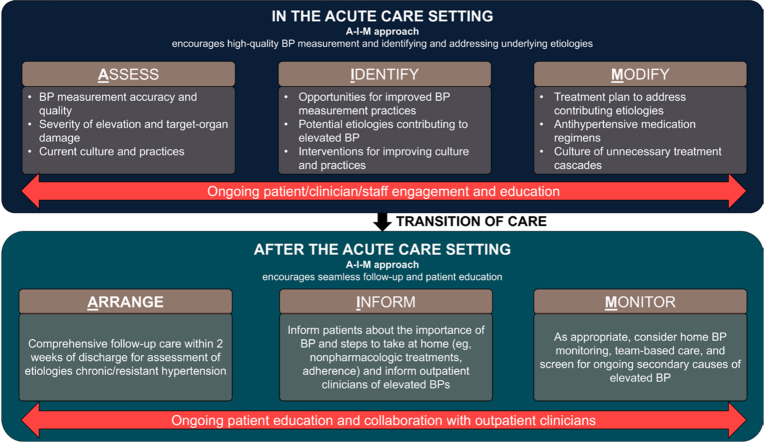

Elevation in blood pressure (BP) is one of the most common clinical entities in the inpatient setting, affecting up to 72% of hospitalized patients.^1^ Approximately one-third of these patients will receive an intravenous (IV) antihypertensive during their hospitalization. However, observational studies show that the treatment of asymptomatic BP elevations can cause harm, including hypotension, acute kidney injury, myocardial injury, stroke, and intensive care unit transfer, while not improving cardiovascular outcomes.^2^, ^3^, ^4^ The American Heart Association (AHA) guidelines recommend that IV antihypertensives be used only for hypertensive emergencies, defined as elevations in BP with evidence of acute end-organ damage.^1^ IV hydralazine is of particular concern due to its unpredictable BP-lowering effects and documented association with orthostatic hypotension and reflex tachycardia.^3^

**Take-Home Messages**

• System-level changes such as order set redesign with embedded safety checks and guideline links are highly effective at reducing inappropriate prescribing when paired with targeted education.
• Real-time data-query capabilities enable identification of persistent inappropriate use, guide targeted interventions, and support sustained improvement.
• This approach is scalable and can be applied to other medications and settings, offering a reproducible model for safer, evidence-based inpatient prescribing.

## Project Rationale

From June 2023 to January 2024, a retrospective review of a representative sample of 100 randomly selected cases at Uconn John Dempsey Hospital showed that approximately 69% of IV hydralazine orders were given without evidence of end-organ damage. At the time, the order set for IV hydralazine at our hospital did not contain any safety features and also defaulted to a q6h pro re nata (PRN) dosing schedule. With the retrospective review data, an initiative began with the goal of reducing IV hydralazine use from 69% to under 30% by July 2025. To help achieve this goal, a root cause analysis was performed, which identified lack of provider awareness, outdated order defaults, and a lack of clear guideline support as the most significant issues.

## Project Description

In April 2025, a redesign of the Epic order set for IV hydralazine was launched at our tertiary care center. As part of the new order set, a hard stop was added requiring providers ordering IV hydralazine to select specific guideline-indicated reasons in order to continue. After multiple stakeholder meetings and review of the literature, the indications “severely elevated hypertension in pregnancy and postpartum” and “signs of end-organ damage” were agreed upon as appropriate. An option labeled “other” was also included, but in order to proceed, the prescriber would need to enter a free-text justification for the order. The goal of the hard stop was to reduce reflexive prescribing of IV hydralazine for asymptomatic BP elevations.

To further support clinicians who were considering IV hydralazine, the Epic order set also included a log of the 5 most recent BPs. Links were added to the most recent AHA guidelines regarding IV hydralazine use, so clinicians could view and alternative management options for elevated BP. Lastly, the q6h PRN default was removed, allowing clinicians to more accurately decide if IV hydralazine was necessary for their patient, rather than prescribing it reflexively.

**Figure 1:**
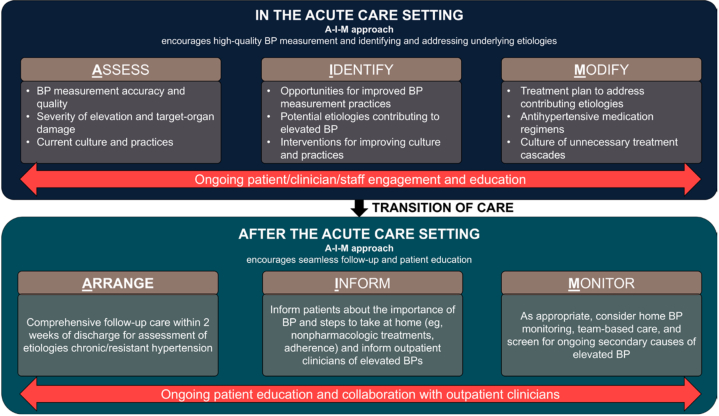
The AIM-AIM Approach Model The figure demonstrates an example model for managing BP in a holistic fashion and provides an alternative approach to managing elevated blood pressure without IV hypertensives. This was incorporated into the Epic order and educational materials. This model was derived from the American Heart Association Scientific Statement.^1^ BP = blood pressure.

In addition to changes to the order, educational seminars were initiated. From March to May 2025, nursing huddles and resident didactic sessions were targeted for brief counseling on current guidelines about the usage of IV hydralazine, as well as safer alternative approaches such as the AHA-recommended “AIM-AIM” approach to asymptomatic BP elevations (Figure 1). To aid with the education sessions, visual aids and flyers were produced and distributed during the meetings. These were also placed on various med-surg floors throughout the hospital.

## Project Deliverables

**Figure 2:**
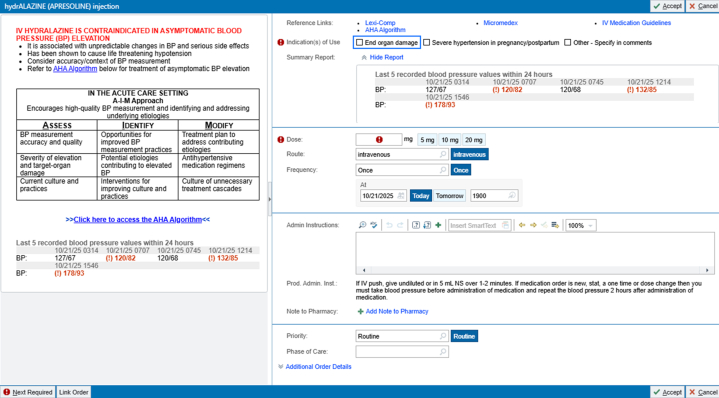
Epic Order Set Modifications The figure shows the modified IV hydralazine order set. In bold red letters, on the top left of the interface, there is a reminder about potential risks of nonindicated IV hydralazine use. There is also the AIM approach model below to help guide decision making. The patient's 5 most recent blood pressures are also displayed. “Indication(s) of Use” represents a hard stop where clinicians must choose the indication for IV hydralazine to prescribe it. The dosage frequency has also been altered to serve a single PRN dose, and not a recurring dose by default. IV = intravenous; PRN = pro re nata.

The initiative led to incorporation of new tools and processes that enhanced patient safety regarding inpatient BP management. The primary deliverable was the permanent redesign of the Epic order (Figure 2) to include mandatory indication selections, links to current AHA-backed inpatient hypertension-management guidelines, a display of the patient's 5 most recent BP measurements, and the removal of the previously incorrect q6h PRN default. The second deliverable was a standardized education curriculum, including visual aid flyers, PowerPoint presentations, and talking points distributed to clinical and nursing teams. The final deliverable created a framework for monitoring prescribing patterns and documented indications for IV hydralazine. Using Epic SlicerDicer, this framework allows continuous safety oversight and feedback regarding the impact of these interventions.

## Results

**Figure 3:**
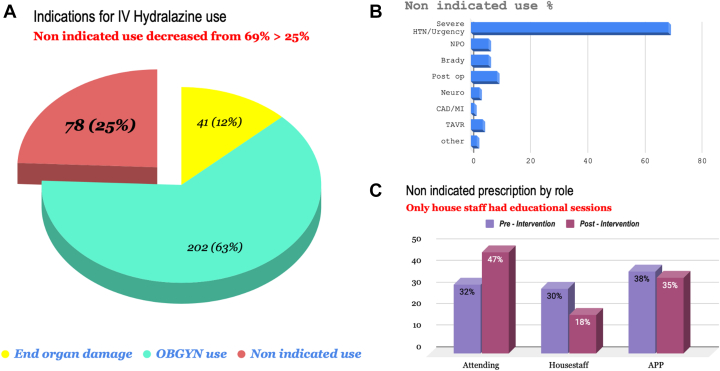
Trends of IV Hydralazine Use Post Intervention (A) Documentation of all IV hydralazine use including severe HTN in pregnancy, end-organ damage, and nonindicated use. (B) Percentage breakdown of orders for each nonindicated use of IV hydralazine, with hypertensive urgency being the most common, followed by postop patients. (C) Changes in nonindicated use by provider role (attending, housestaff, APP) from preintervention (purple) to postintervention (magenta). APP = advanced practice provider; CAD = coronary artery disease; HTN = hypertension; IV = intravenous; MI = myocardial infarction; NPO = nil per os; TAVR = transcatheter aortic valve replacement.

Baseline sampled data revealed that IV hydralazine was used 69% of the time without an appropriate indication. After the implementation of the revamped Epic order set, inappropriate use decreased to 25% (78 out of a total of 321 orders) between April and July 2025 (Figure 3A). Of the remaining nonindicated use, the most common free-text indication continued to be “hypertensive urgency/elevated blood pressure.” Other common entries were “NPO patients with bradycardia” and “postoperative patients” (Figure 3B). Education was most effective in conjunction with the order set, as housestaff showed greater proportional change in prescribing patterns than attendings or advanced practice providers (Figure 3C).

## Discussion

Our study demonstrated that Epic order set changes combined with targeted education can drastically reduce nonindicated IV hydralazine use. The success of this initiative began with a baseline assessment of the problem and engagement of stakeholders early on to collaboratively define indications and develop solutions. This revealed the following problems with the Epic order: lack of required indications, no recent BP display, default PRN dosing, and no embedded guidelines. All of these were addressed with a single redesign. Education helped reinforce these changes and was most effective as a supplement to the redesign rather than as a standalone intervention.

The initiative demonstrated that nonindicated IV hydralazine use was largely driven by reflexive clinician behaviors and unclear guidelines surrounding asymptomatic BP elevations. Although the order set reduced inappropriate use significantly, issues persisted in specific clinical contexts, highlighting the need for continued education and monitoring. A key advantage of the redesign is the ability to query free-text indications and ordering details, helping identify errors and their rationale. This will directly guide our next steps, including targeted interventions with evidence-based alternatives for common nonindicated scenarios (eg, severe hypertension, postoperative hypertension, nil per os with bradycardia), while highlighting departments or roles where education will have the most impact.

This model of order set modifications combined with targeted education has immense potential for broader application. It could be applied to other medications, such as labetalol, which is the next target for our team. More broadly, this strategy could be replicated in any hospital using Epic or a similar electronic medical record. This provides an effective and scalable method for safer, evidence-based prescribing.

## Conclusions

Implementing system-level changes through Epic order set modifications, combined with targeted education, can significantly reduce nonindicated prescribing of potentially harmful medications. Early stakeholder engagement, comprehensive problem assessment, and addressing multiple workflow barriers in a single redesign were key to success. Embedding required indications, decision-support tools, and data-query capabilities not only reduced inappropriate IV hydralazine use but also created a framework for ongoing monitoring, targeted feedback, and replication for other medications and institutions.

## Funding Support and Author Disclosures

The authors have reported that they have no relationships relevant to the contents of this paper to disclose.

## References

[1] Bress A.P., Anderson T.S., Flack J.M., American Heart Association Council on Hypertension; Council on Cardiovascular and Stroke Nursing; and Council on Clinical Cardiology (2024). The management of elevated blood pressure in the acute care setting: a scientific statement from the American Heart Association. Hypertension.

[2] Wilson L.M., Herzig S.J., Steinman M.A. (2024). Management of inpatient elevated blood pressures: a systematic review of clinical practice guidelines. Ann Intern Med.

[3] Campbell P., Baker W.L., Bendel S.D., White W.B. (2011). Intravenous hydralazine for blood pressure management in the hospitalized patient: its use is often unjustified. J Am Soc Hypertens.

[4] Peixoto A.J. (2019). Acute severe hypertension. N Engl J Med.

